# Distemper in a Dolphin

**DOI:** 10.3201/eid1312.070309

**Published:** 2007-12

**Authors:** Peter Wohlsein, Christina Puff, Mihaela Kreutzer, Ursula Siebert, Wolfgang Baumgärtner

**Affiliations:** * University of Veterinary Medicine, Hannover, Germany; † Christian-Albrechts-University, Buesum, Germany

**Keywords:** Dolphin, distemper, morbillivirus, encephalitis, letter

**To the Editor:** Deaths caused by new members of the genus *Morbillivirus*, family Paramyxoviridae (*1*), have occurred in recent decades among phocine and cetacean species, particularly harbor seals (*Phoca vitulina*) in 1988 (*2*) and 2002 (*3*). Endangered Mediterranean striped dolphins (*Stenella coeruleoalba*) died in 1990 and 1991 (*4*), and common dolphins (*Delphinus delphis ponticus*) from the Black Sea died in 1994 because of infection with dolphin morbillivirus (DMV) (*5*). A similar virus caused deaths in bottlenose dolphins (*Tursiops truncatus*) in the Gulf of Mexico from 1987 through 1994 (*6*). Closely related morbilliviruses caused deaths in harbor porpoises (*Phocoena phocoena*) in European waters in 1988 (*7*) (*Porpoise morbillivirus*) and endangered Mediterranean monk seals (*Monachus monachus*) in 1997 (*8*) (*Monk seal morbillivirus*). After these epidemics, the viruses disappeared and no marine or terrestrial reservoirs have been identified.

In January 2007, a moribund, subadult, white-beaked dolphin (*Lagenorhynchus albirostris*) was found stranded on the North Friesian coast of Germany. The animal was humanely killed and a complete necropsy was performed. The main lesion was a nonsuppurative meningoencephalitis with neuronal degeneration and few eosinophilic cytoplasmic inclusion bodies characteristic of a viral disease. Lungs showed suppurative and interstitial pneumonia. Paraffin-embedded sections of brain were examined for morbillivirus antigen by using an immunoperoxidase technique. We used various monoclonal antibodies that recognize different morbilliviruses. Tissues from a seal infected with phocine distemper virus and a dog with canine distemper were used as positive controls. Tissues from a white-beaked dolphin that underwent an autopsy in 2006 were used as negative controls. In the diseased dolphin, morbillivirus antigen was found exclusively in neurons and glial cells of the brain (Figure, panel A).

**Figure:**
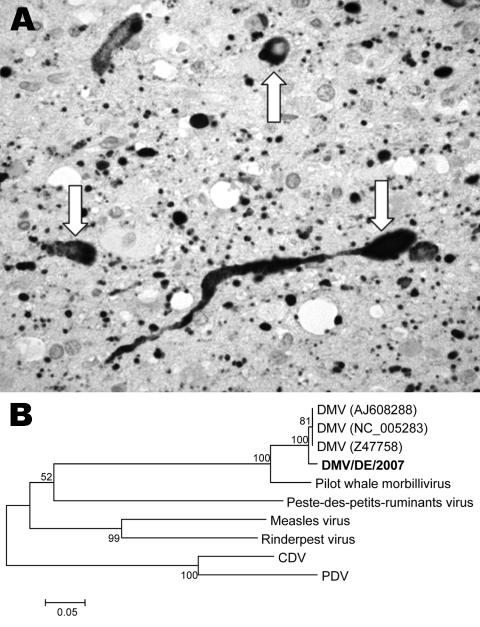
A) Immunohistologic demonstration of morbillivirus antigen in cytoplasm and nuclei of neurons (arrows) and glial cells in the brain of a white-beaked dolphin, using a monoclonal antibody (GenWay, San Diego, CA, USA) against nucleoprotein of canine distemper virus (CDV)/phocine distemper virus (PDV) visible as numerous black dots (magnification ×630). B) Unrooted neighbor-joining phylogenetic tree constructed by using 353 nt from the gene coding for the morbillivirus phosphoprotein. Alignments were calculated with ClustalX version 1.83 (http://bips.u-strasbourg.fr/fr/Documentation/ClustalX). Bootstrapping (values indicated in %) was performed with 1,000 replicates using MEGA 3.1 software (www.megasoftware.net/mega.html). The new isolate from this study is shown in **boldface**. The following sequences were included: dolphin morbillivirus (DMV) (GenBank accession nos. NC_005283, Z47758, AJ608288), pilot whale morbillivirus (AF200817), Peste-des-petits-ruminants virus (NC_006383), measles virus (NC_001498), Rinderpest virus (NC_006296), CDV (NC_001921), and PDV (D10371). Scale bar shows nucleotide substitutions per site.

Frozen tissue samples and blood were examined for morbillivirus nucleic acid by reverse transcription–PCR with a set of universal morbillivirus primers that are specific for highly conserved regions of virus nucleoprotein (N) (*9*) and phosphoprotein (P) (*10*). A 457-bp amplicon of the P gene (GenBank accession no. EF451565) and a 287-bp amplicon of the N gene (GenBank accession no. EF469546) were detected in brain tissue. Our isolate, DMV/DE/2007, showed homologies of 99% with the N gene and 98% with the P gene of DMV isolated from Mediterranean striped dolphins. Phylogenetic analysis showed that isolate DMV/DE/2007 is closely related to DMV (Figure, panel B), porpoise morbillivirus, and monk seal morbillivirus (*8*).

Histologic changes in the dolphin resembled those of distemper in seals (*3*), porpoises (*7*), and other dolphins (*4*–*6*). Identification of morbillivirus antigen in diseased tissues and isolation of genome fragments of a morbillivirus provide conclusive evidence for a primary etiologic role of this virus. Sequencing of the virus and phylogenetic comparison showed that the virus is closely related to previously described dolphin morbillivirus and porpoise and monk seal morbilliviruses (*8*). To our knowledge, this is the first report of morbillivirus infection in a white-beaked dolphin in German waters and in a marine mammal since the last epidemic among harbor seals in northern Europe in 2002. Isolation of DMV has not been reported since 1994.

Our findings indicate that DMV is still circulating in some marine mammals. Similar to infections in terrestrial hosts, morbillivirus infections may occur in marine mammals in cycles without overt clinical disease in susceptible animals, as documented for harbor seals (*2*,*3*). Serum samples collected from 1995 through 1999 from cetacean species in various regions were positive for DMV, but porpoises and striped dolphins showed a decrease in humoral immunity, making them vulnerable to new epidemics. No data exist on seroprevalence of morbillivirus-specific antibodies in white-beaked dolphins. We do not know how the dolphin contracted the infection and whether this remains an isolated case or the beginning of a new zoonosis.

White-beaked dolphins are found in moderate and subarctic waters of the Atlantic Ocean between the eastern coast of North America and northern Europe. They may migrate hundreds of kilometers within days. Therefore, these dolphins may play a role as a reservoir and vector for this morbillivirus, which is infectious for harbor porpoises, bottlenose dolphins, and other cetacean species (*10*). The reappearance of a morbillivirus represents a serious threat to susceptible marine mammals in northern European and American waters, with potentially devastating consequences and possibly the beginning of a new epidemic.
